# Effect of periodontal therapy on the oral microbiome and lung function: an intervention study

**DOI:** 10.3389/fcimb.2026.1725666

**Published:** 2026-03-18

**Authors:** Anders Røsland, Hesham Amin, Stein Atle Lie, Andrei Malinovschi, Dagmar F. Bunæs, Randi J. Bertelsen

**Affiliations:** 1 Department of Clinical Dentistry, Section of Periodontics, University Of Bergen, Bergen, Norway; 2 Department of Clinical Dentistry, Centre for Translational Oral Research (TOR), University of Bergen, Bergen, Norway; 3 Department of Clinical Science, University Of Bergen, Bergen, Norway; 4 Department of Medical Sciences, Clinical Physiology, Uppsala University, Uppsala, Sweden; 5 Oral Health Centre of Expertise in Western Norway, Bergen, Norway

**Keywords:** airway resistance, forced oscillation technique, oral-lung axis, periodontal therapy, periodontitis, shotgun metagenomics, plaque subgingival microbiome

## Abstract

**Introduction:**

The oral cavity harbors over 700 bacterial species, and disruption of this balance can lead to periodontitis, which has been linked to systemic conditions including respiratory disease.

**Methods:**

In this longitudinal clinical trial, 57 never-smoking adults with stage I–II periodontitis underwent full-mouth periodontal disinfection. Airway resistance and subgingival plaque sampling (analyzed by shotgun metagenomics) was measured at baseline and six weeks after therapy.

**Results:**

Periodontal treatment significantly improved clinical periodontal parameters, and was associated with reductions in airway resistance. Microbiome analysis showed a shift from periodontitis-associated taxa, including *Prevotella*, *Porphyromonas*, and *Tannerella*, toward health-associated species such as *Actinomyces oris*, and *Rothia dentocariosa*. Higher airway resistance was associated with a greater relative abundance of periodontitis-associated bacteria.

**Discussion:**

Together, findings suggest that periodontal therapy promotes a healthier oral microbiome and is associated with improved lung function in non-smokers with no prior lung disease.

## 1 Introduction and objectives

The oral cavity is the second most diverse microbiome in the human body, housing over 700 different bacterial species (Avila et al., 2009). Despite the dense and varied microflora, a delicate balance between the host’s immune responses and oral microbes prevents acute infections of the oral tissues (Zaura et al., 2014). However, when this balance is disrupted, it can lead to various oral and systemic diseases (Gao et al., 2018; Bourgeois et al., 2019).

Periodontitis is one of the most prevalent diseases globally (GBD 2015 Disease and Injury Incidence and Prevalence Collaborators, 2016) and is a leading cause of tooth loss over a lifetime (Kassebaum et al., 2014). As periodontitis progresses, subgingival biofilms adhere to the tooth root surface, leading to inflammation in the surrounding periodontal tissues (Kolenbrander et al., 2010). The shift in the subgingival microbiota is driven by dysbiosis (Hajishengallis and Lamont, 2021) with a predominance of disease-associated pathobionts, including *Porphyromonas*, *Treponema*, and *Tannerella (*Abusleme et al., 2021). Unlike other conditions where microbial diversity decreases, periodontitis is characterized by increased bacterial diversity and richness (Costalonga and Herzberg, 2014). Periodontal therapy aims to restore a healthy balance in the oral microbiome by reducing harmful bacteria and promoting the growth of beneficial microbes (Teles et al., 2006; Colombo et al., 2012). The 2020 clinical guidelines from the European Federation of Periodontology recommend a structured, stepwise approach to treating periodontitis including effective self-performed dental plaque control (Sanz et al., 2020).

Poor oral health has significant systemic effects, with numerous studies linking periodontitis to various systemic diseases (Tonetti et al., 2013), including respiratory disorders (Gaeckle et al., 2020; Molina et al., 2023). For a long time, the presence of oral bacteria in lung samples was believed to be due to contamination during bronchoscopy. However, advanced sampling techniques, such as protected brushing, have disproven this possibility, confirming that healthy lungs harbor a microbial community and that oral bacteria are the primary source of microbes in the healthy adult lung (Blainey et al., 2012; Dickson et al., 2017). These findings underscore the close relationship between the oral cavity and the lungs.

**Figure 1:**
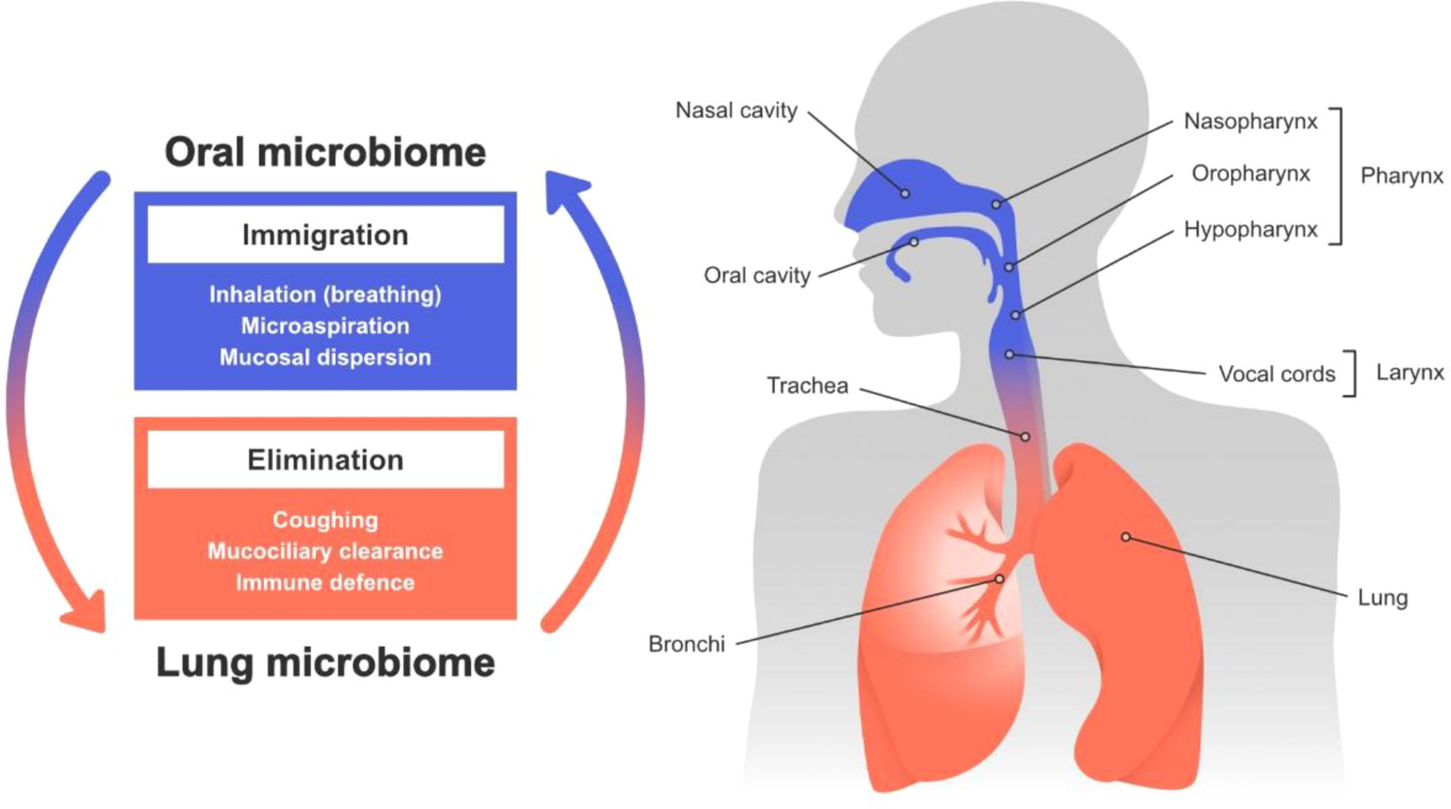
Illustrates the anatomical and ecological relationship between the oropharynx and respiratory tract, highlighting processes that shape the lung microbiome. Immigration pathways include inhalation, microaspiration, and mucosal dispersion, while elimination mechanisms, such as coughing, mucociliary clearance, and immune defenses, maintain microbial balance. This conceptual framework provides biological context for the hypothesized oral–respiratory interactions examined in the present study. Figure design: Joana C. Carvalho^©^.

Oral bacteria may enter the lungs through micro-aspiration and mucosal dispersal, however a constant natural elimination process (illustrated conceptually in **Figure 1**), together with regional growth conditions, determines which microorganisms ultimately become established (Dickson and Huffnagle, 2015). This provides one biological explanation linking periodontitis with respiratory diseases; whereby micro-aspiration of periodontitis-associated bacteria and their components into the lower airways, may promote airway inflammation (Imai et al., 2021). In addition to direct aspiration, systemic inflammatory pathways may also contribute to this association (Hajishengallis, 2015). Virulence factors produced by periodontal pathogens, such as *Porphyromonas gingivalis*, including gingipains and lipopolysaccharide (LPS), can stimulate systemic immune activation and modulate inflammatory signaling, potentially influencing airway inflammatory status. Periodontitis and impaired lung function also share common risk factors (Cullinan et al., 2009; Zeng et al., 2012), and are both associated with systemic inflammation (Shaaban et al., 2006; Thyagarajan et al., 2006; Rasmussen et al., 2009; Yoshii et al., 2009; Gläser et al., 2012). Because airway resistance may reflect inflammatory changes within the peripheral airways (Burgel et al., 2009), techniques capable of detecting subtle alterations in airway mechanics may be particularly informative in this context. The Forced Oscillation Technique (FOT) is a sensitive method for detecting lung function impairment, even when spirometry results appear normal (Qvarnström et al., 2023). A recent study demonstrated a relationship between abnormal FOT measurements and systemic inflammation, even in the presence of normal spirometry (Färdig et al., 2024). Therefore, it is plausible that FOT could be associated with the systemic low-grade inflammation induced by periodontitis-associated bacteria.

We have previously demonstrated that periodontitis therapy significantly improves clinical periodontal parameters and was associated with improvements in lung function in adults with no medical conditions other than periodontitis (Røsland et al., 2025). While the impact of mechanical periodontal treatment on clinical periodontal parameters is well-documented (Cobb, 2002), the interplay between these clinical effects, dynamics of bacterial compositions in the subgingival biofilm and respiratory health remains unexplored. Therefore, this study aims to investigate the relationship between changes in the subgingival oral microbiome induced by periodontal therapy and changes in lung function in an otherwise healthy, never-smoking population.

## 2 Materials and methods

### 2.1 Study design

The present study is a single-blind, prospective, longitudinal clinical trial designed to evaluate change in oral microbiome and lung function following periodontal therapy. Flow-chart of enrolment, therapy, data collection, dropout timepoints, and sample size calculations are described previously (Røsland et al., 2025). The trial was conducted in accordance with the STROBE (Strengthening the Reporting of Observational Studies in Epidemiology) guidelines to ensure comprehensive and transparent reporting.

### 2.2 Study participants

The study included 57 never-smoking, healthy participants with non-severe periodontitis and high dental plaque- and bleeding percentage (≥50%). Details on enrolment, inclusion- and exclusion criteria are previously described (Røsland et al., 2025). Oral examination and periodontal treatment were performed by one intra-calibrated operator (AR) at the Department of Clinical Dentistry, University of Bergen from April 2021 through June 2023.

### 2.3 Data collection

The data collection comprised lung function tests, questionnaires, and subgingival plaque sampling together with a full-mouth periodontal charting. Lung function assessment and questionnaire completion were conducted on the same day, prior to the dental examination. For the present analysis, we included data from two time points: baseline (T0) and follow-up (T1), which occurred six weeks after periodontal therapy.

#### 2.3.1 Lung function assessment

Lung function data, the primary outcome, were collected by trained fieldworkers at the Research Unit for Health Surveys (RUHS), University of Bergen. Participants underwent lung function testing using both standard spirometry and the Forced Oscillation Technique (FOT), as previously described (Røsland et al., 2025). Spirometry evaluates lung function by measuring airflow and lung volumes during forced maneuvers, such as forced vital capacity (FVC) and forced expiratory volume in one second (FEV_1_). It primarily identifies large airways’ obstruction aiding in the diagnostic work-up of respiratory conditions. FOT provides additional insights by detecting small airway dysfunction and early-stage airway obstruction, which may not be detected by spirometry. Key indices from FOT are respiratory system resistance (Rrs) and reactance (Xrs). Rrs reflects airway patency and the opposition to airflow within the airways, whereas Xrs reflects the elastic and inertive properties of the respiratory system (King et al., 2020). Elevated Rrs values indicate greater airway obstruction, leading to increased airflow resistance and making breathing more difficult for the patient.

#### 2.3.2 Periodontal variables

Dental and medical data were documented in the dental records during the periodontal examination. The radiographic examination included bitewing radiographs, supplemented with periapical radiographs when necessary. The clinical examination involved a full-mouth assessment of periodontal pocket depth (PD) and clinical attachment loss (CAL) using a periodontal probe with 1 mm gradings (Hu-Friedy PCPUNC157). PD was measured at six sites per tooth, recording the distance from the gingival margin to the pocket base, and CAL as the distance from the cementum–enamel junction to the pocket depth (excluding third molars). Gingival inflammation and dental plaque were assessed by recording the percentage of sites with bleeding on probing (BoP) and plaque index (PI). BoP was registered as the presence of bleeding (Ainamo and Bay, 1975), and plaque as detectable supragingival plaque (O’Leary et al., 1972) using a disclosure agent (Curaprox, PCA 260). Periodontitis were diagnosed according to the 2017 World Workshop on the Classification of Periodontal and Peri-Implant Diseases and Conditions (Tonetti et al., 2018).

#### 2.3.3 Subgingival plaque sampling

All patients underwent a clinical evaluation during a pre-screening examination conducted before the baseline examination. During this pre-screening, patients were assessed based on clinical inclusion criteria, and pathological pockets were identified and recorded. By pre-identifying the deepest pockets, we aimed to avoid contamination of sampling sites from the full-mouth probing conducted at baseline. At baseline, subgingival plaque samples were collected first from the deepest pocket in each quadrant using sterile curettes (Hu-Friedy, Chicago, IL, USA). If multiple sites exhibited equally deep probing depths, the most accessible site was selected. Subgingival samples were taken from the same sites at both baseline and follow-up. The selected sites were isolated with cotton rolls, supragingival plaque was removed, and subgingival plaque was collected with a single stroke of the curette from the pocket base. Plaque samples were pooled in CryoPure tubes (1.8 ml) by immersing the curette in sterile phosphate-buffered saline (PBS) and were immediately stored at −80 °C until further analysis.

#### 2.3.4 Other patient related assessments

Anthropometric data and questionnaire data were collected during the clinical examinations at Research Unit for Health Surveys (RUHS). Further details can be found in the Additional file 1.

### 2.4 Periodontal therapy

Periodontal therapy was conducted according to the S3-level clinical practice guidelines from the European Federation of Periodontology (Sanz et al., 2020) using a full-mouth disinfection approach as previously described (Røsland et al., 2025).

### 2.5 Bacterial microbiota profiling of subgingival plaque samples

Bacterial DNA extraction and microbiota profiling were conducted by Clinical Microbiomics (Copenhagen, Denmark). The DNA extraction of the 114 sub-gingival samples from the participants collected at baseline (n=57) and follow-up (n=57), was performed with the NucleoSpin 96 Soil (Macherey-Nagel) kit. The analyses also included negative and positive controls (see the supplementary appendix for details). The library construction was based on the NEBNext Ultra Library Prep Kit for Illumina (New England Biolabs) and sequenced using 2 × 150 bp paired-end sequencing on an Illumina platform. For profiling of the raw reads the Clinical Microbiomics Human Microbiome Profiler was used, which identifies and quantifies Metagenomic Species (MGS) (Nielsen et al., 2014). The quality of the taxonomic profiles of the participant samples after background removal was very good as the profiles were based on average on 1.07 M reads mapping to the MGS signature genes (minimum of 26550 reads). See the Additional file 1 for more details.

### 2.6 Statistical analysis

Summary statistics were presented as frequencies for categorical variables and means with standard deviations (SD) for continuous variables. Differences between groups over time were tested in the mixed effects model with the use of an interaction term between groups and time. P-values (two sided) less than 0.05 were considered statistically significant. All statistical analyses assessing changes in lung function and periodontal parameters were performed using mixed effects models in Stata 17.0 (StataCorp, College Station, TX, USA).

Alpha diversity was evaluated using the Shannon index, which reflects both species richness and evenness, along with bacterial count, measured by the observed number of taxa identified through metagenomic species (MGS). Differences in alpha diversity were evaluated using the Wilcoxon rank-sum test and visualized by box plots. Beta diversity, reflecting the variation in bacterial diversity among samples, was assessed via the Bray–Curtis dissimilarity metric, with significance determined by PERMANOVA (Anderson, 2001), and the results were visualized with a Principal Coordinate Analysis (PCoA) plot. To determine which taxa were differentially represented between time points and groups, we conducted a differential abundance analysis at the species level using Analysis of Compositions of Microbiomes with Bias Correction (ANCOM-BC2) (Lin and Peddada, 2020) using the pseudo-count sensitivity analysis with Holm-Boferroni correction for multiple testing. The model was adjusted for age, sex, and BMI. This approach is designed to identify differentially abundant microbes between groups while accounting for covariates. Data analyses were performed using R (version 4.4.1). Figures were generated using the ggplot2 (version 3.5.1) R package, and the ampvis2 package (version 2.8.9) for heatmaps. To investigate associations between microbial abundances and airway resistance variables, we utilized the Multivariable Association with Linear Models 2 (MaAsLin2) (Mallick et al., 2021) package in R. The model included fixed effects for airway resistance variable (R_5_), sex, body mass index (BMI), and age. Random effects were included for repeated measurements to account for inter-individual variability. The microbial abundance data were normalized using total sum scaling (TSS) and log-transformed prior to analysis. MaAsLin2 results were visualized with bar plots. All R scripts, processed datasets, and analysis workflows are publicly available at our GitHub repository (https://github.com/University-of-Bergen/oral-microbiome-periodontal-study).

In our previously reported analyses of this cohort, periodontitis therapy was associated with significant improvements in airway resistance, with reductions observed as early as six weeks and sustained over the 12-month follow-up period (Røsland et al., 2025).These findings provided the clinical rationale for further stratified microbiome analyses in the present study (Røsland et al., 2025). Resistance at 5 Hz (R_5_), a key indicator of both central and peripheral airway resistance and an overall measure of airway obstruction, consistently declined, reaching statistical significance by the three-month follow-up (p=0.001). R5 was selected for stratification as it reflects total airway resistance, encompassing both central and peripheral airway compartments, whereas higher frequencies (e.g., R11 and R19) primarily represent central airway mechanics (Røsland et al., 2025). To objectively assess whether certain patients experienced a more pronounced treatment effect we performed a stratified analysis evaluating differences in oscillometry outcomes between patients above and below the R_5_ median at baseline (absolute value of R_5_). No participants had a baseline R_5_ value equal to the median. The analysis revealed that patients with baseline R_5_ values above the median (indicating decreased airway patency compared to those with R_5_ values below the median) greater improvements in lung function (**Supplementary Figure 1**; **Additional file 1**). To explore whether these improvements could be attributed to differences in bacterial profiles, we stratified patients into two groups for the analysis presented in this paper: (I) those with baseline R_5_ above the median (3.06 cm H_2_O.L^-1^.s^-1^) and (II) those with baseline R_5_ below the median.

### 2.7 Ethics statement

Ethical approval was obtained from the Regional Committee for Medical and Health Research Ethics in Western Norway (approval no. #94605) and the trial was registered at ClinicalTrials.gov (NCT04781153) on February 19, 2021, prior to inclusion. All participants provided written informed consent.

## 3 Results

### 3.1 Baseline characteristics

Baseline characteristics of the study population stratified by sex are summarized in **Table 1**. A total of 57 participants were included in the trial, with a mean age of 36 years, and a female majority (60%). Stage I periodontitis was diagnosed in 17.5% of participants, and 82.5% had Stage II periodontitis. All participants exhibited normal lung function measurement at baseline.

**Table 1:** Baseline characteristics of study population stratified by sex.

Baseline characteristics of study population		Sex	
Variables	Total	Female	Male
Participants	*n* = 57	34 (60%)	23 (40%)
Age (SD)	35.9 (6.1)	36.5 (5.9)	35.0 (6.4)
Married or cohabitant	35 (61.4%)	21 (60.0%)	14 (40.0%)
Education at University Level	48 (84.2%)	30 (62.5%)	18 (37.5%)
Employed	47 (82.5%)	29 (61.7%)	18 (38.3%)
Body mass index in kg/m^2^ (SD)	27.0 (5.1)	27.3 (5.3)	26.7 (4.7)
Height in cm (SD)	172.0 (9.9)	165.9 (6.3)	181.0 (6.3)
Weight in kg (SD)	80.4 (18.2)	75.5 (17.6)	87.7 (16.8)
Severity of periodontitis			
Stage I	10 (17.5%)	7 (70.0%)	3 (30.0%)
Stage II	47 (82.5%)	27 (57.4%)	20 (42.6%)
Exercise			
Once a week or less	17 (30.9%)	8 (47.1%)	9 (52.9%)
2–3 times per week	23 (41.8%)	16 (69.6%)	7 (30.4%)
Almost every day	15 (27.3%)	9 (60.0%)	6 (40.0%)
%predicted spirometry variables			
%predicted FEV_1_ (SD)	93.90 (10.90)	95.80 (10.93)	91.10 (10.53)
%predicted FVC (SD)	98.50 (11.73)	100.60 (10.90)	95.35 (12.50)
Absolute values of oscillometry variables			
R_5_ (SD)	3.20 (1.10)	3.52 (1.15)	2.70 (0.84)
R_11_ (SD)	3.00 (0.86)	3.30 (0.87)	2.55 (0.64)
R_19_ (SD)	2.80 (0.73)	3.10 (0.70)	2.33 (0.50)
Periodontal variables			
BoP in % (SD)	63.60 (14.23)	63.10 (14.50)	64.31 (14.11)
PI in % (SD)	62.02 (10.52)	61.70 (10.50)	62.54 (10.80)
PD in mm (SD)	2.49 (0.19)	2.49 (0.20)	2.49 (0.19)
CAL in mm (SD)	2.72 (0.19)	2.70 (0.19)	2.74 (0.19)

Data are presented as *n* (%) or mean and standard deviations (SD). Percent predicted values were calculated from the 2012 Global Lung function Initiative. FEV_1_, forced expiratory volume in the first second; FVC, forced vital capacity; R_5_, airway resistance measured at 5Hz; R_11_, airway resistance measured at 11Hz; R_19_, airway resistance measured at 19Hz; BoP, bleeding on probing; PI, plaque index; PD, periodontal pocket depth; CAL, clinical attachment loss.

### 3.2 Treatment effect on microbiome composition from baseline (T0) to follow-up (T1)

The full-mouth disinfection therapy administered to the participants resulted in significant improvements in all clinical periodontal parameters (p<0.001) at the six-week follow-up, detailed in **Supplementary Table 1**, **Additional file 1**.

#### 3.2.1 Alpha and beta diversity

**Figure 2:**
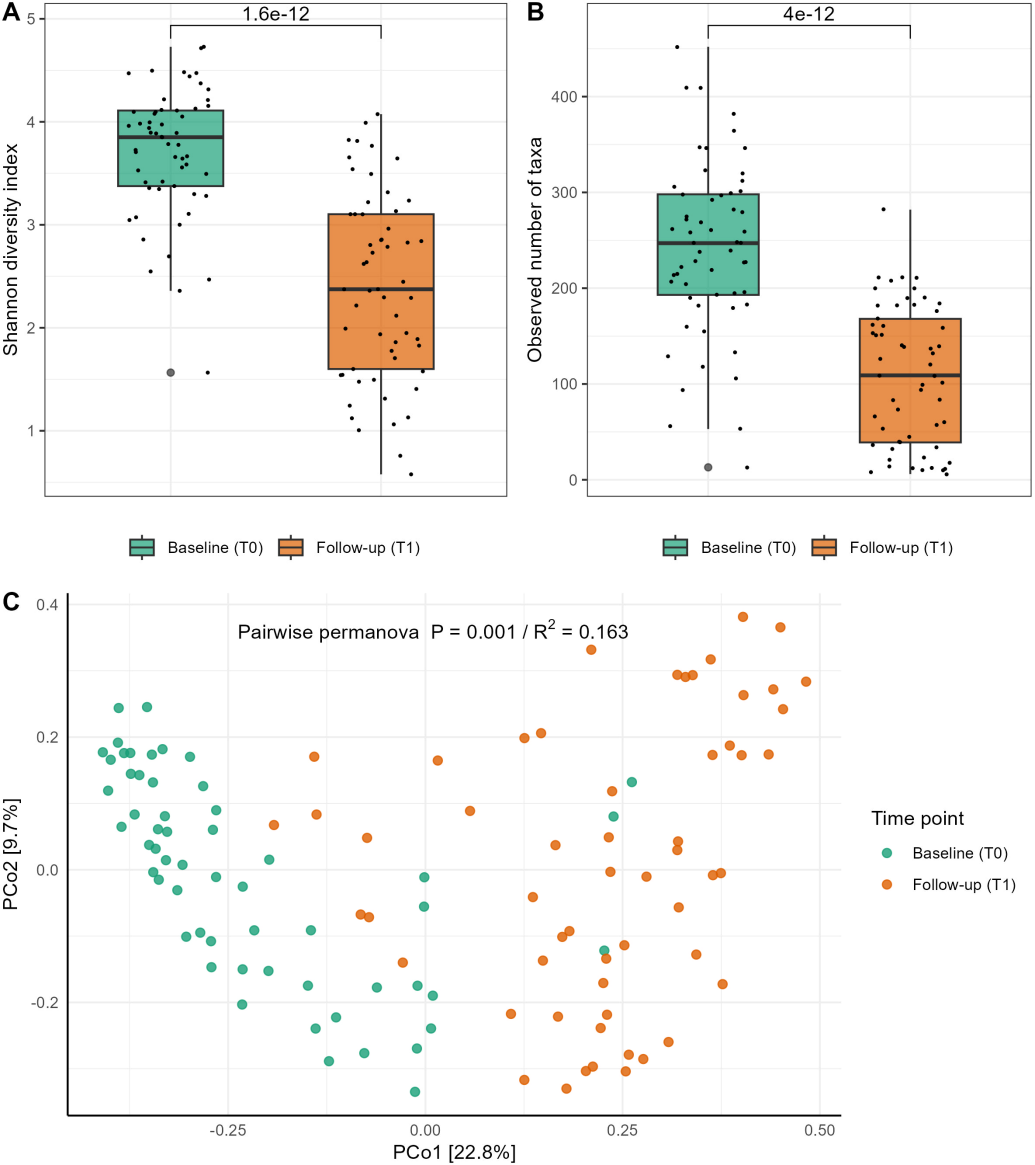
Difference in alpha and beta diversity of the subgingival microbiome collected from the deepest pockets at baseline (T0) and from the same pockets at follow-up (T1) six weeks after periodontal therapy. **(A)** Shannon Diversity Index; **(B)** Observed Number of taxa/MGS (Metagenomic species); results from Wilcoxon rank-sum test p<0.001 for both Shannon and MGS. **(C)** Principal coordinate analysis (PCoA) plot for comparison between baseline and follow-up samples (Bray–Curtis dissimilarity metric); pairwise PERMANOVA results, p = 0.001; R^2^ represents proportion variance in the data explained by time points.

The alpha diversity of the deep pocket subgingival microbiome showed a significant decrease from baseline to follow-up across both diversity indices (p<0.001) (**Figures 2A, B**). Similarly, beta diversity analysis demonstrated significant shifts in the microbial composition following periodontal therapy (p<0.001). The R² value indicated that 16.3% of the variance in microbial community composition could be attributed to the time point (T0 *vs*. T1), highlighting that the observed changes in microbiome composition were significantly associated with the intervention (**Figure 2C**).

#### 3.2.2 Relative abundant bacterial genera and species

**Figure 3:**
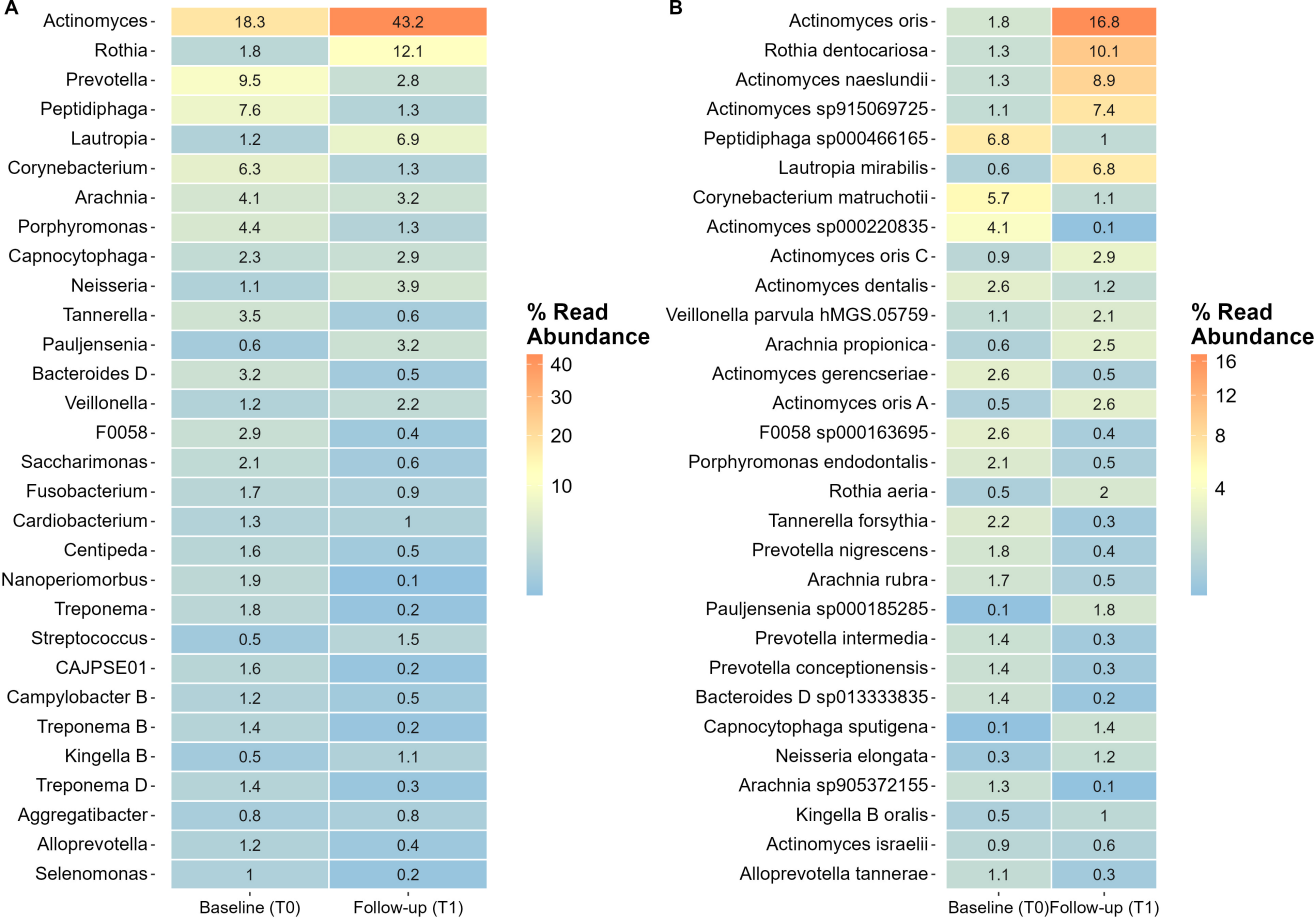
Showing the relative abundance (% contribution) of the 30 most prevalent bacteria. Heat map **(A)** bacterial genera, and heat map **(B)** bacterial species. T0, baseline, and T1, follow-up six weeks following periodontal therapy.

The heatmap revealed that baseline subgingival plaque samples were predominantly composed of bacteria from the genera *Actinomyces*, *Prevotella*, *Peptidiphaga*, *Corynebacterium*, and *Porphyromonas* accounting >46% of the sequences. At follow-up, the most prevalent genera were *Actinomyces*, *Rothia*, and *Lautropia*, collectively accounting for over 60% of the sequences (**Figure 3A**).

At the species level, *Peptidiphaga* sp*000466165*, *Corynebacterium matruchotii*, and *Actinomyces* sp*000220835* were the most abundant at baseline, comprising more than 16% of all bacterial species. At follow-up, the most notable increases were observed in *Actinomyces oris* (16.8% *vs*. 1.8%), *Rothia dentocariosa* (10.1% *vs*. 1.3%), *Actinomyces naeslundii* (8.9% *vs*. 1.3%), and *Actinomyces* sp*915006725* (7.4% *vs*. 1.1%) (**Figure 3B**).

The ANCOM-BC2 model identified statistically significant changes in the abundance of 95 bacterial species from baseline to follow-up, with 13 species absent at follow-up (classified as structural zeros at T1) (p<0.05). *Actinomyces oris* and *Pauljensenia odontolytica A*, exhibited the most substantial increases at follow-up with a Log Fold Change (LFC)>4. In contrast, *Olsenella F* sp*001189515* and *Peptidiphaga* sp*000466165* displayed the largest decreases, with LFCs of -3.3 and -3.1, respectively. Additionally, taxa associated with periodontitis, including *Porphyromonas* spp., *Treponema* spp., and *Prevotella* spp., were significantly reduced or not detected at follow-up indicating their complete elimination following the intervention. Further details on the ANCOM-BC2 results are provided in the **Additional file 2**.

### 3.3 Associations between microbial abundances and airway resistance

**Figure 4:**
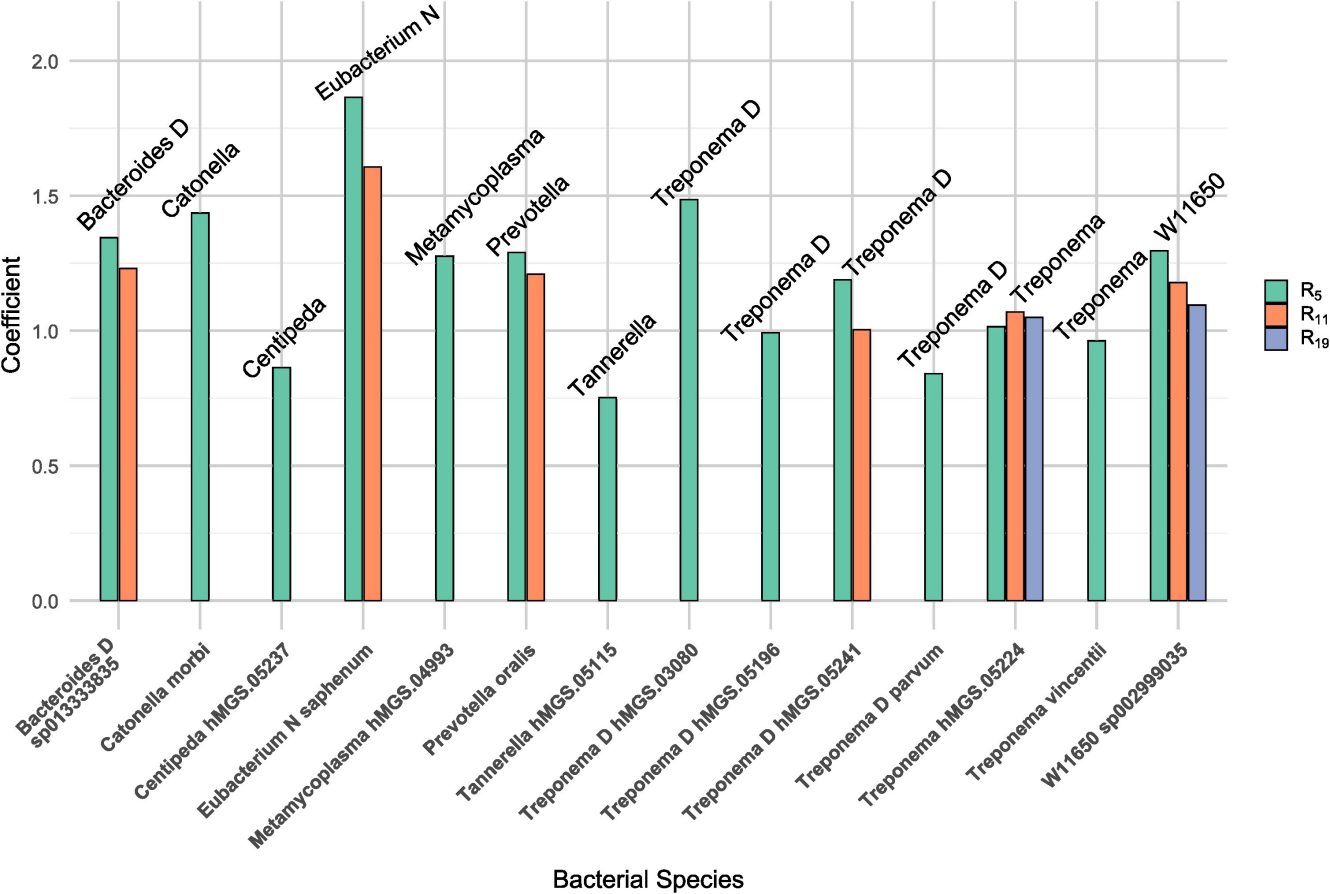
Presents the bacterial species that were significantly associated with increasing airway resistance. Each bar represents a species, and the height of the bar corresponds to its coefficient. The color-coded bars indicate associations with airway resistance at different frequencies: green for 5Hz (R_5_), orange for 11Hz (R_11_), and blue for 19Hz (R_19_). The analysis was conducted using the MaAsLin2 package in R.

To investigate the longitudinal associations between airway resistance and bacterial species abundances, the MaAsLin2 package was used to assess how absolute values of airway resistance are related to microbial abundances, while accounting for repeated measures. The analysis identified statistically significant positive associations between 14 bacterial species and R_5_, six bacterial species and R_11_, and two species was associated with an increase of R_19_ (**Figure 4**). The interpretation of the coefficients in the figure suggests that for each one-unit increase in airway resistance (a continuous variable), the abundance of the corresponding bacterial species increases by the magnitude of the respective coefficient.

Higher airway resistance was associated with an increased abundance of several periodontitis-associated bacteria, including *Prevotella* spp., *Tannerella* spp., and *Treponema* spp. Specifically, an increase in R_5_ was associated with a higher abundance of all bacterial species shown in the plot, with the strongest association observed for *Eubacterium N saphenum*. Similarly, an increase in R_11_ was linked to a higher abundance of six bacterial species, with *Eubacterium N saphenum* showing the strongest association, followed by *Bacteroides D* sp*013333835*. Finally, an increase in R_19_ was significantly associated with a higher abundance of *W11650* sp*002999035* and *Treponema hMGS.05224*. For details on the results, please see **Additional file 3**.

### 3.4 Treatment effect on microbiome composition stratified by individuals with baseline measurements above and below the median of R_5_

#### 3.4.1 Alpha- and beta diversity before therapy (at T0)

At baseline, no statistically significant difference was observed in alpha- or beta diversity between individuals grouped by the R_5_ median (**Supplementary Figure 2**; **Additional file 1**).

#### 3.4.2 Alpha and beta diversity from baseline (T0) to follow-up (T1)

**Figure 5:**
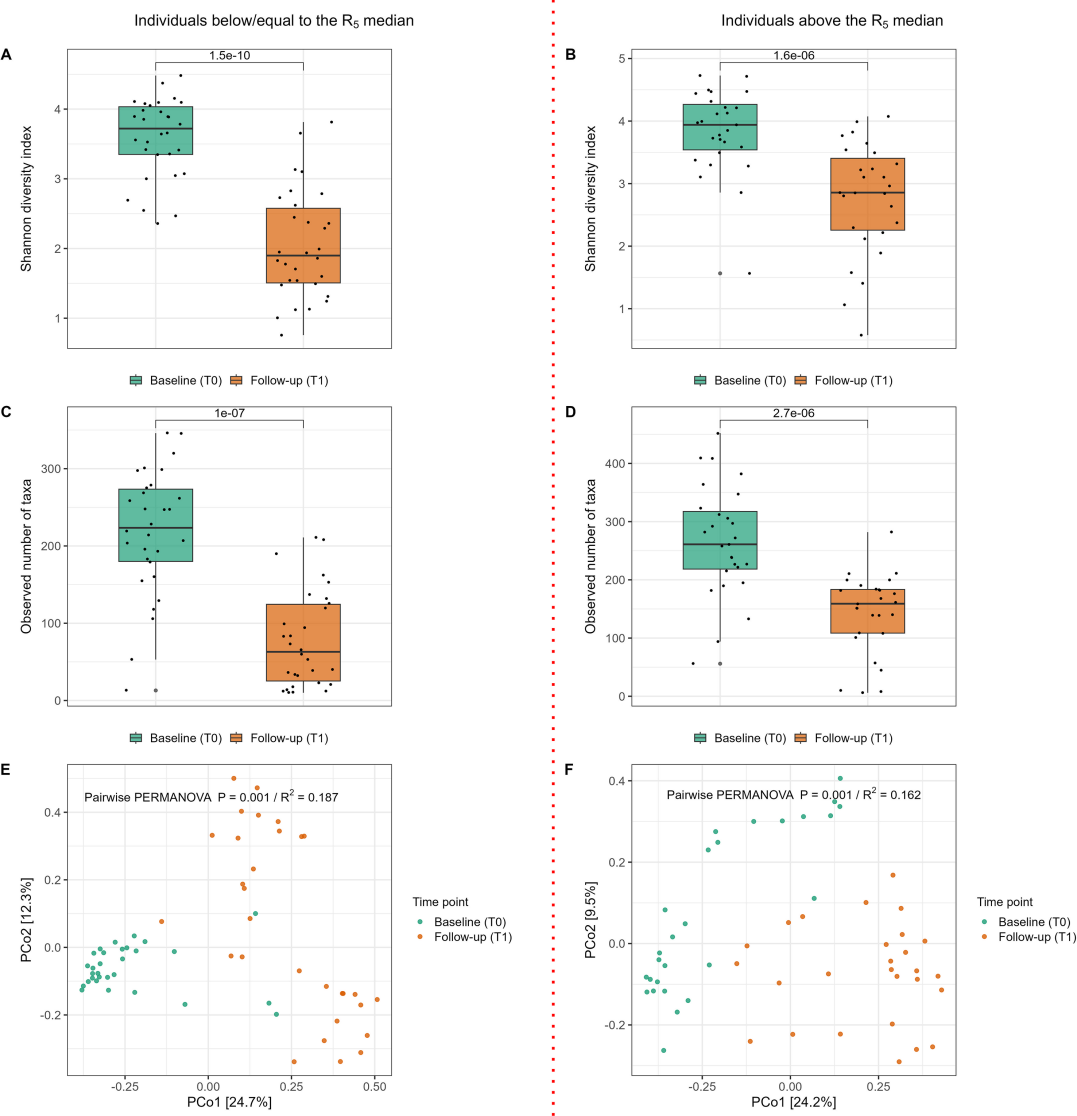
Alpha and beta diversity at baseline (T0) and follow-up (T1) stratified by R_5_ median. **(A)** Shannon Diversity Index among individuals with R_5_ below the median; **(B)** Shannon Diversity Index among individuals with R_5_ above the median; **(C)** Observed Number of MGS (Metagenomic species) among individuals below the R_5_ median; **(D)** Observed Number of MGS among individuals above the R_5_ median. Results from Wilcoxon rank-sum test p<0.001. **(E)** Principal coordinate analysis (PCoA) plot (Bray–Curtis dissimilaritymetric) among individuals with R_5_ values below the median; **(F)** Principal coordinate analysis (PCoA) plot (Bray–Curtis dissimilaritymetric) among individuals above the R_5_ median; pairwise PERMANOVA results, p = 0.001; R^2^ represents proportion variance in the data explained by the grouping factors.

**Figure 3** show alpha and beta diversity from T0 to T1 among individuals below baseline R_5_ median (**Figures 5A, C, E**), and among the individuals above R_5_ median (**Figures B, D, F**). Statistically significant differences in all diversity indices were observed between timepoints and groups following periodontal therapy (p<0.05). Notably, no statistically significant differences were observed between the two groups in clinical periodontal parameters at either T0 or T1 (**Supplementary Figure 3**; **Additional file 1**).

#### 3.4.3 Alpha and beta diversity following therapy (at T1)

At follow-up (T1), statistically significant differences in both alpha and beta diversity were observed between individuals with baseline R_5_ values above the median and those with values below. Alpha diversity analysis revealed significantly higher diversity in individuals with R_5_ values above the median (p<0.05). Similarly, beta diversity analysis demonstrated significant differences between the two R_5_ median strata (p=0.033) (**Supplementary Figure 4**; **Additional file 1**).

#### 3.4.4 Relative abundance of bacterial genera and species at baseline (T0) and follow-up (T1)

**Figure 6:**
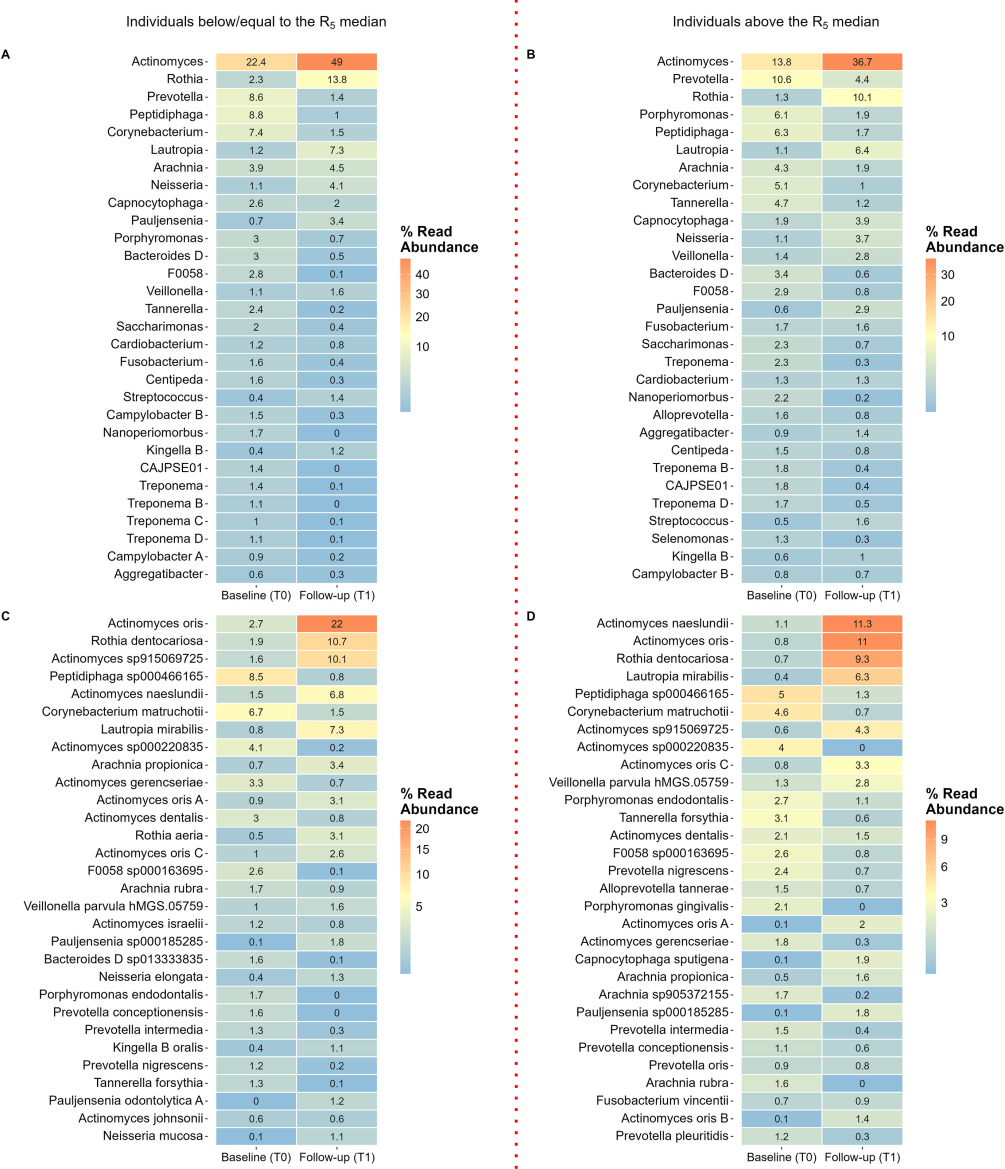
Relative abundance (% contribution) of the 30 most prevalent bacterial genera **(A, B)** and species **(C, D)** at baseline (T0) and at follow-up (T1) stratified by individuals above and below the R_5_ median. Heat map **(A)** relative abundance of bacterial genera among patients below the R_5_ median. Heat map **(B)** relative abundance of bacterial species among individuals above the R_5_ median. Heat map **(C)** relative abundance of bacterial species among patients below the R_5_ median. Heat map **(D)** relative abundance of bacterial species among individuals above the R_5_ median. The capital letter behind the genus and species names refers to distinct sequence variants within the same genus or species but not identified with taxonomic name in the database.

At baseline (T0), the bacterial genera with the highest abundances were *Actinomyces*, *Prevotella*, and *Peptidiphaga*, collectively accounting for over 30% of the microbiota in all individuals. The *Porphyromonas* genus was more prevalent in individuals with the highest airway resistance (R_5_ values above the median), accounting for 6.1% of the microbiota compared to 3% in those with less airway resistance (**Figures 6A, B**). At species level, the most abundant taxa were *Peptidiphaga* sp*000466165*, *Corynebacterium matruchotii*, and *Actinomyces* sp*000220835*, collectively accounting for 13% of the microbiota in individuals with high resistance and 19% in those with a lower resistance (**Figures 6C, D**).

Individuals above the R_5_ median exhibited a higher relative abundance of periodontitis-associated genera at baseline, including *Prevotella, Porphyromonas*, and *Tannerella*, and a lower relative abundance of genera associated with oral health, such as *Actinomyces* and *Rothia*, compared to those at or below the R_5_ median (**Figures 6A, B**). Moreover, *Porphyromonas endodontalis* was 1.5 times more abundant, and *Tannerella forsythia* was more than 2 times more abundant in individuals with high resistance. In contrast, *P. gingivalis* was not among the 30 most abundant species in individuals below the R_5_ median (**Figure 6C, D**).

At follow-up (T1), the most prevalent bacterial species in both groups included *Actinomyces naeslundii*, *A. oris*, *Rothia dentocariosa*, *Lautropia mirabilis*, and *A.* sp*915069725*. Among these, *A. oris* and *A.* sp*915069725* were more abundant in individuals below the R_5_ median, accounting for 21.6% versus 11% and 10% versus 4.3%, respectively. Both strata exhibited an overall reduction in the relative abundance of periodontitis-associated species, including *T. forsythia, Prevotella intermedia*, and *Porphyromonas endodontalis*. Among patients with R_5_ values above the median, the relative abundance of *P. gingivalis* decreased from 2.1% to 0 after periodontal therapy (**Figures 6C, D**).

To identify differentially abundant bacterial species from baseline to follow-up among individuals stratified by the R_5_ median, the ANCOM-BC2 model was applied. Among participants above the R_5_ median, the analysis detected statistically significant changes in the abundance of 48 bacterial species (p<0.05), with six species absent at follow-up (classified as structural zeros at T1) and one species absent at baseline (structural zero at T0). In individuals below the R_5_ median, 46 species showed significant differential abundance, with 11 species classified as structural zeros at T1 and one species absent at T0. Details of the ANCOM-BC2 results are provided in the Additional file 2.

At follow-up, among individuals above the R_5_ median, the greatest decrease in abundance was observed in *T. forsythia* (LFC < -3.0), followed by *Olsenella* sp*001189515* (LFC = -2.9) and *Corynebacterium matruchotii* (LFC = -1.95). The most pronounced increase was observed in *Actinomyces oris* (LFC = 4.3), followed by *Pauljensenia* sp*000185285* and *Actinomyces* sp*915069725*, both with an LFC of 3.8.

A different pattern emerged among individuals at or below the R_5_ median, where the most significant decrease in abundance was observed in *Neisseria sicca A* (LFC = -15.8), followed by *Nanosynbacter* sp*905373315* (LFC = -9.1) and *Parvimonas* sp*000214475* (LFC = -7.3). Conversely, the species showing the greatest increase in abundance were *Capnocytophaga hMGS.01622* (LFC = 22.6), *Haemophilus D parainfluenzae* (LFC = 6.8), and *Pauljensenia odontolytica A* (LFC = 5.8).

## 4 Discussion

This clinical trial provides new insights into the relationship between periodontal therapy- associated changes in the subgingival oral microbiome and changes in airway resistance. Our findings demonstrate that periodontal therapy not only reduces bacterial diversity but also increases the relative abundance of health-associated taxa in the subgingival microbiota. In parallel, and consistent with our previously reported findings in this cohort (Røsland et al., 2025), airway resistance declined following periodontal therapy, particularly in individuals with elevated baseline R_5_ values. Although reductions in airway resistance were observed, the clinical meaningfulness of these changes should be interpreted cautiously, as established oscillometric thresholds are primarily defined for diseased populations rather than healthy individuals. Nevertheless, the persistence of these reductions over the 12-month follow-up suggests a sustained physiological shift (Røsland et al., 2025).

To further explore whether baseline airway resistance was associated with distinct microbial signatures, we stratified participants according to the R5 median and compared their subgingival microbiome profiles. Individuals with high airway resistance at baseline exhibited a higher relative abundance of periodontitis-associated genera compared to those with low airway resistance. These findings may support a potential association between impaired lung function and the typical anaerobic, proteolytic pathogenic bacteria residing in the deep pockets, and which are more frequently associated with systemic inflammation. Conversely, individuals with lower airway resistance initially had a higher abundance of genera associated with oral health, such as *Actinomyces* and *Rothia*. Following therapy, both groups showed a significant reduction in the relative abundance of key periodontitis-associated genera, alongside an increase in commensal genera.

Oral bacteria are hypothesized to enter the lower respiratory tract via microaspiration, representing a biologically plausible pathway linking oral dysbiosis with respiratory physiology. In the present study, higher airway resistance was associated with an increased relative abundance of periodontitis-associated taxa, including *Prevotella*, *Tannerella*, and *Treponema*, supporting the hypothesis that subgingival dysbiosis may be linked to airway mechanics. Epidemiological studies have consistently shown associations between periodontitis and impaired lung health, including airflow obstruction (Røsland et al., 2024), airflow limitation (Holtfreter et al., 2013), and reduced spirometry indices such as FEV_1_ (Lee et al., 2020). Research has revealed that bacterial communities in healthy lungs significantly overlap with those in the oral cavity, albeit at lower concentrations (Bassis et al., 2015), suggesting a possible link between oral and pulmonary microbiota. Emerging evidence highlights potential mechanisms by which oral bacteria influence pulmonary function and contribute to respiratory diseases. For instance, periodontopathic bacteria have been implicated in the development of aspiration pneumonia and chronic obstructive pulmonary disease (COPD) (Scannapieco, 1999). Studies comparing periodontal status between pneumonia and non-pneumonia patients show that individuals with periodontal disease are more prone to developing pneumonia (Gomes-Filho et al., 2014). Additionally, negative associations have been observed between the relative abundance of subgingival periodontitis-associated taxa, such as *P. gingivalis*, and lung function in COPD patients (Tan et al., 2019). In line with these observations, periodontal therapy in our cohort was associated with both a reduction in periodontitis-associated species and improvements in airway resistance, suggesting that modification of the oral microbial burden may influence respiratory physiology even in individuals without overt lung disease. Previous interventional studies in COPD, populations similarly indicate that reducing oral inflammation may positively impact respiratory outcomes (Kucukcoskun et al., 2013; Zhou et al., 2014; Sundh et al., 2021).

In addition to direct microaspiration, indirect systemic inflammatory pathways may also contribute to the associations observed in the present study. Periodontitis is characterized by an enrichment of Gram-negative anaerobic taxa including *P. gingivalis*, *T. forsythia*, and *P. intermedia (*Hajishengallis, 2015), several of which were detected at baseline and were more abundant among individuals with higher airway resistance. These organisms produce a range of virulence factors, including LPS, gingipains, and outer membrane vesicles, which can stimulate systemic immune activation, promote epithelial invasion, and modulate host inflammatory signaling (Zhang et al., 2025; Jia et al., 2019; Schäffer and Andrukhov, 2024). Such mechanisms may contribute to low-grade systemic inflammation and altered airway inflammatory tone, providing a biologically plausible explanation for the observed associations between subgingival dysbiosis and airway resistance. In our cohort, periodontal therapy markedly reduced the relative abundance of these periodontitis-associated species, alongside observed improvements in airway resistance, suggesting that modification of the oral inflammatory burden may influence respiratory physiology even in otherwise healthy individuals. While the present study provides robust taxonomic characterization of therapy-induced microbial shifts, functional pathway-level profiling was not performed. Future analyses leveraging the shotgun metagenomic dataset, including assessment of LPS biosynthesis pathways, proteolytic virulence factors, and inflammatory signaling signatures, may further clarify the mechanistic links between oral microbial shifts and respiratory physiology.

Beyond the direct and systemic mechanisms discussed above, the ecological restructuring of the subgingival microbiome following therapy may represent an additional pathway linking periodontal treatment to improved airway resistance. Periodontal treatment, specifically mechanical supra- and subgingival scaling and root planning, induces ecological changes in the subgingival environment by reducing pathogenic bacteria and promoting the growth of beneficial microbes (Socransky et al., 2013). In the present study, therapy was associated with a significant reduction of key periodontitis-associated species, including *T. forsythia*, *P. intermedia*, and *P. gingivalis*, alongside an increased relative abundance of health-associated bacterial species, such as *Actinomyces* spp. (*A. oris, A. naeslundii*), *R. dentocariosa*, and *L. mirabilis*. These findings underscore the marked contrast between the dysbiotic microbial profile at baseline and the post-therapy microbiome composition. One of the primary benefits of a resident beneficial microbiota is its ability to prevent colonization by exogenous and pathogenic bacteria, a phenomenon known as “colonization resistance”. This protective effect arises from mechanisms, including the formation of structured biofilms that act as physical barriers to harmful microbes, the production of antimicrobial substances like hydrogen peroxide and bacteriocins, and the inhibition of pathogen growth through competitive exclusion and nutrient depletion (Marsh, 2000).

In the analyses comparing individuals with high and low airway resistance, no statistically significant differences in alpha or beta diversity were observed between the groups at baseline. However, individuals with high resistance showed a higher relative abundance of periodontitis-associated genera and a lower relative abundance of periodontal health-associated genera compared to those below the R_5_ median. Following periodontal therapy, microbial profiles in both groups converged toward a more health-associated composition. These findings are consistent with previous studies that report a transition to a more health-associated microbiome following periodontal treatment (Shi et al., 2015). Interestingly, individuals above the R_5_ median demonstrated higher bacterial diversity after therapy compared to those below the median. In periodontal disease, increased subgingival alpha diversity reflects the expanded ecological niches created by deep periodontal pockets, allowing colonization by anaerobic and opportunistic taxa. Following therapy, pocket reduction removes these niches, resulting in decreased diversity and a shift toward a more health-associated microbial community (Liu et al., 2012). Beta diversity analysis also showed significant differences in microbial community composition between the two groups. Although the six weeks may not fully capture the long-term stabilization of the oral microbiome and respiratory outcomes, these findings suggest that individuals with higher baseline airway resistance may harbor a more dysbiotic subgingival microbiome in the deep periodontal pockets, rendering it more responsive to intervention. This observation supports the hypothesis that a greater inflammatory or microbial burden provides a larger window for therapeutic improvement.

The multivariable linear regression analysis (MaAsLin) revealed significant positive associations between airway resistance and multiple bacterial genera known to contribute to periodontal inflammation and dysbiosis, including *Prevotella* spp., *Tannerella* spp., and *Treponema* spp (Socransky et al., 1998; Meuric et al., 2017). Notably, the strongest positive association between airway resistance and bacterial abundance was observed for *Eubacterium nodatum saphenum*, a species previously isolated from periodontal pockets (Uematsu et al., 1993). These findings support the observed enrichment of periodontitis-associated taxa among individuals with higher baseline airway resistance in our cohort. While evidence on the direct impact of these specific bacteria on respiratory health remains limited, similar interacting consortia found in the oral cavity also exist in the lower respiratory tract with lower abundance and diversity (Bassis et al., 2015). Given that the oropharynx serves as the primary source of the lung microbiome in healthy individuals, it is plausible that the aspiration of oral pathogens into the lungs may contribute to respiratory disease.

Future analyses framed within classical periodontal pathogen complex models may further enhance clinical interpretation. In particular, evaluating aggregate abundance of the red complex (*P. gingivalis*, *T. forsythia*, *T. denticola*) and the orange complex taxa (Suzuki et al., 2013; Gambin et al., 2021), often described as ecological “bridging” organisms, could provide additional insight into microbial succession and treatment-induced ecological shifts. Such approaches may help determine whether coordinated pathogenic consortia, rather than individual taxa alone, drive the associations observed between subgingival dysbiosis and airway resistance. Additionally, integration of pathogen complex–based approaches with virulence factor profiling, including proteolytic enzymes and lipopolysaccharide pathways, may help clarify mechanistic links between periodontal dysbiosis and respiratory physiology.

### 4.1 Strengths and limitations

This study possesses several strengths. Although extensive research has explored the role of oral bacteria in respiratory diseases, evidence linking the oral microbiota to lung function in the absence of overt disease remains limited. Uniquely, this study examines the longitudinal relationship between periodontal therapy, the subgingival microbiome, and lung function in never-smoking adults with early-stage periodontitis without preexisting lung disorders. By including healthy, never-smoking individuals aged 25–45 who had reached their maximum lung capacity (Eisner et al., 2010), we aimed to minimize potential confounding factors. Lung function naturally increases from birth until young adulthood (around 18–20 years in males and slightly later in females), after which it reaches a plateau before gradually declining with age (Eisner et al., 2010). By selecting never-smoking participants who had already attained peak lung function, we minimized the effects of age-related lung development and avoided the confounding influence of smoking, a strong risk factor for both periodontitis and respiratory disease (Hyman and Reid, 2004). While this selective design strengthens internal validity, it may limit generalizability, as early-stage periodontal disease is the far most common state of the disease, even among individuals without underlying medical conditions.

The analysis of subgingival plaque samples using shotgun metagenomics represents a significant strength of the study. This robust and comprehensive method enables precise identification of bacterial taxa within the microbiome, offering a detailed understanding of how periodontal therapy influences the subgingival microbiome and its potential links to respiratory health. Furthermore, the study employed objective lung function assessments using FOT measurements, which are particularly valuable for detecting airflow obstruction in the peripheral airways. Unlike traditional spirometry, FOT is more sensitive to early and subtle changes in lung function, providing deeper insights into the potential respiratory benefits of periodontal therapy. In contrast to the present study, previous research on oral health and lung function has exclusively used spirometry for lung function assessment. Another strength is that all participants received the same intervention following a well-established clinical protocol by one calibrated operator (AR), ensuring consistency in treatment and minimizing variability.

Several limitations should be acknowledged in this study. First, the absence of a control group limits the ability to definitively attribute lung function improvements to the intervention, as natural variations or placebo effects cannot be ruled out. This study design was made based on ethical considerations, given the progressive nature of periodontitis. Withholding standard periodontal treatment, which is well-established in clinical practice, would have posed ethical concerns due to the potential risk of harm to participants. To address this, a pre-post study design was implemented, allowing participants to serve as their own controls, with baseline measurements used to assess the effects of therapy on the outcome variables. Second, subgingival plaque sampling was limited to the deepest periodontal pocket at each time point, which may bias the microbiome toward diseased niches and limit representativeness of the overall subgingival ecosystem (Ge et al., 2013). Accordingly, the findings should be interpreted as reflecting microbiota from inflamed periodontal sites rather than the entire subgingival ecosystem. However, bacteria residing in the deep pockets are more frequently associated with systemic inflammation, and have more access to the bloodstream through ulcerated pocket epithelium and thus are more biologically plausible drivers of changes in lung function. Additionally, it is important to acknowledge that the current microbiome indices used in periodontal research primarily focus on specific genera or species, providing a limited view of the overall microbial community. This approach may overlook the complex interactions and synergistic effects within the broader subgingival ecosystem. Future studies should incorporate analyses of the dynamic and complex nature of the oral microbiome to avoid underestimating the contributions of less abundant taxa, which may still play significant roles in maintaining oral health or driving disease progression. Another consideration is the potential presence of unaccounted confounders, such as variations in diet or environmental exposures, that may have influenced both microbiome composition and lung function.

Lastly, the relatively short follow-up period of six weeks may not have been sufficient to capture the full extent of microbiome shifts and lung function improvements or to assess their long-term stability. As we have previously reported (Røsland et al., 2025), airway resistance continues to improve over the twelve-month study period. Therefore, we speculate that longer follow-up could offer valuable insights into sustained changes in microbiome composition. However, subgingival samples from these later time points have not been analyzed, which limits our ability to explore these potential long-term effects in the present study.

In conclusion, this study offers valuable insights into the previously unexplored interplay between periodontal therapy, the oral microbiome, and respiratory health. Periodontal therapy induces notable changes in bacterial diversity, shifting the subgingival microbiome in the periodontal pockets toward a profile dominated by health-associated taxa and this effect is more pronounced in individuals with decreased airways patency. These findings underscore the importance of adopting personalized treatment strategies that account for individual microbial profiles to optimize clinical outcomes. Further research, incorporating longer follow-up periods and more refined analytical methods, including assessments of the dynamic and complex nature of the oral microbiome, is essential to deepen our understanding of the intricate relationship between microbial factors and respiratory health outcomes.

## Data Availability

The datasets presented in this study can be found in online repositories. The names of the repository/repositories and accession number(s) can be found below: https://www.ebi.ac.uk/ena, PRJEB89245.

## Glossary

ANCOM-BC2: analysis of compositions of microbiomes with bias correction 2.0
AR: first author and dental operator/examiner
BMI: body mass index
BoP: bleeding on probing
CAL: clinical attachment loss
COPD: chronic obstructive pulmonary disease
FEV_1_: forced expiratory volume in the first second
FOT: forced oscillation technique
FVC: forced vital capacity
MaAsLin2: multivariable association with linear models, version 2.0. An “R”
MGS: Metagenomic species
PBS: phosphate-buffered saline
PCoA: Principal Coordinate Analysis
PD: probing pocket depth
PI: plaque index
Rrs: respiratory system resistance
RUHS: Research Unit for Health Surveys
R_5_: the resistance measured at 5Hz
R_11_: the resistance measured at 11Hz
R_19_: the resistance measured at 19Hz
STROBE: Strengthening the Reporting of Observational Studies in Epidemiology
Xrs: respiratory system reactance
