# Supplementary material for: Effect of periodontal therapy on the oral microbiome and lung function: an intervention study
Source: Front Cell Infect Microbiol. 2026 Mar 18;16:1725666. doi: 10.3389/fcimb.2026.1725666 (PMC13038898; doi:10.3389/fcimb.2026.1725666)
Supplement: Supplementary file 1 [file Supplementaryfile1.docx]

**Additional file 1: The impact of periodontal therapy on the oral microbiome and lung function: an intervention study**

Anders Røsland^1,2^, Hesham Amin^3^, Stein Atle Lie^1,2^, Andrei Malinovschi^5^, Dagmar F Bunæs^1,2^, Randi J. Bertelsen^3,4^

^1^University Of Bergen, Department of Clinical Dentistry, Section of Periodontics, Bergen, Norway. ^2^University of Bergen, Department of Clinical Dentistry, Centre for Translational Oral Research (TOR), Bergen, Norway. ^3^University Of Bergen, Department of Clinical Science, Bergen, Norway. ^4^Oral health centre of expertise in Western Norway, Bergen, Norway. ^5^Uppsala University, Department of Medical Sciences, Clinical Physiology, Uppsala, Sweden.

Corresponding author: Anders Røsland, Department of Clinical Dentistry, University of Bergen, Årstadveien 19, 5009 Bergen, Norway, telephone: +47 934 05 709, e-mail: [anders.rosland@uib.no](mailto:anders.rosland@uib.no)

**Contents**

[Methods 2](#_Toc198037012)

[Results 4](#_Toc198037013)

[Supplementary figure 1 4](#_Toc198037014)

[Supplementary table 1 5](#_Toc198037015)

[Supplementary figure 2 6](#_Toc198037016)

[Supplementary figure 3 7](#_Toc198037017)

[Supplementary figure 4 8](#_Toc198037018)

[References 9](#_Toc198037019)

# Methods

*Other patient related assessment*

Height (cm) and weight (kg) were measured, and body mass index (BMI) was calculated as weight/height^2^ (kg/m^2^). A questionnaire was used to record socioeconomic status, self-perceived oral and respiratory health, physical activity, and use of antibiotics. For questionnaire distribution and data collection, the web-based platform EasyTrial.net (EasyTrial ApS, Denmark) was used.

*Bacterial microbiota profiling of subgingival plaque samples*

The samples yielded very good sequencing data with an average of more than 1 million reads mapping to the MGS signature genes per participant sample. The microbiome profiling with the Clinical Microbiomics Human Microbiome Reference HMR05 gene catalog with an average of more than 1 million reads mapping to the MGS signature genes per participant sample.

The DNA extraction was performed with the NucleoSpin 96 Soil (Macherey-Nagel) kit, and the analyses also included one negative control per batch of samples and a positive (mock) control (ZymoBIOMICS Microbial Community Standard (Zymo Research), and ten PBS control samples were provided to identify background signal coming from lab reagents and sample handling.

Before sequencing, the quality of the DNA samples was evaluated using agarose gel electrophoresis and the quantity of the DNA was evaluated by Qubit 2.0 fluorometer quantitation.

The fragmented genomic DNA (fragments of about 350 bp) was used for library construction using NEBNext Ultra Library Prep Kit for Illumina (New England Biolabs). The library was sequenced using 2 × 150 bp paired-end sequencing on an Illumina platform. Sequencing data were obtained from all participant samples, with an average of 36.48 million (M) read pairs per sample. The ten PBS samples had less than 1.04 M read pairs on average.

For profiling of the raw reads we used Clinical Microbiomics Human Microbiome Profiler that identifies and quantifies Metagenomic Species (MGS)[1] based on the Clinical Microbiomics Human Microbiome Reference HMR05 gene catalog with 25,761,278 genes. On average, 16.7 M read pairs remained per participant sample after filtering low-quality and host reads (we refer to these remaining read pairs as high quality nonhost (HQNH) read pairs) (an average of 54% of the read pairs of the participant samples mapped to the human genome). On average, 82% of the HQNH read pairs were mapped to the gene catalog resulting in an average of 13.7 M mapped read pairs per participant sample.

*Background signaling filtering*

An MGS was kept as a biological signal if the maximum raw count in a sample was at least 4 times as high as the highest count in a negative control. Common background signals, such as *Ralstonia*, *Burkholderia*, and *Bradyrhizobium* species, were removed, as described in [2]. Overall, 37 MGS were identified as background signals leaving 884 MGS considered true biological signal.

The quality of the taxonomic profiles of the participant samples after background removal was very good as the profiles were based on average on 1.07 M reads mapping to the MGS signature genes (minimum of 26550 reads).

# Results

Supplementary figure 1. Changes in oscillometry variables from T0 to T1, stratified by patients with a baseline R_5_>median and those with a baseline R_5_<median.


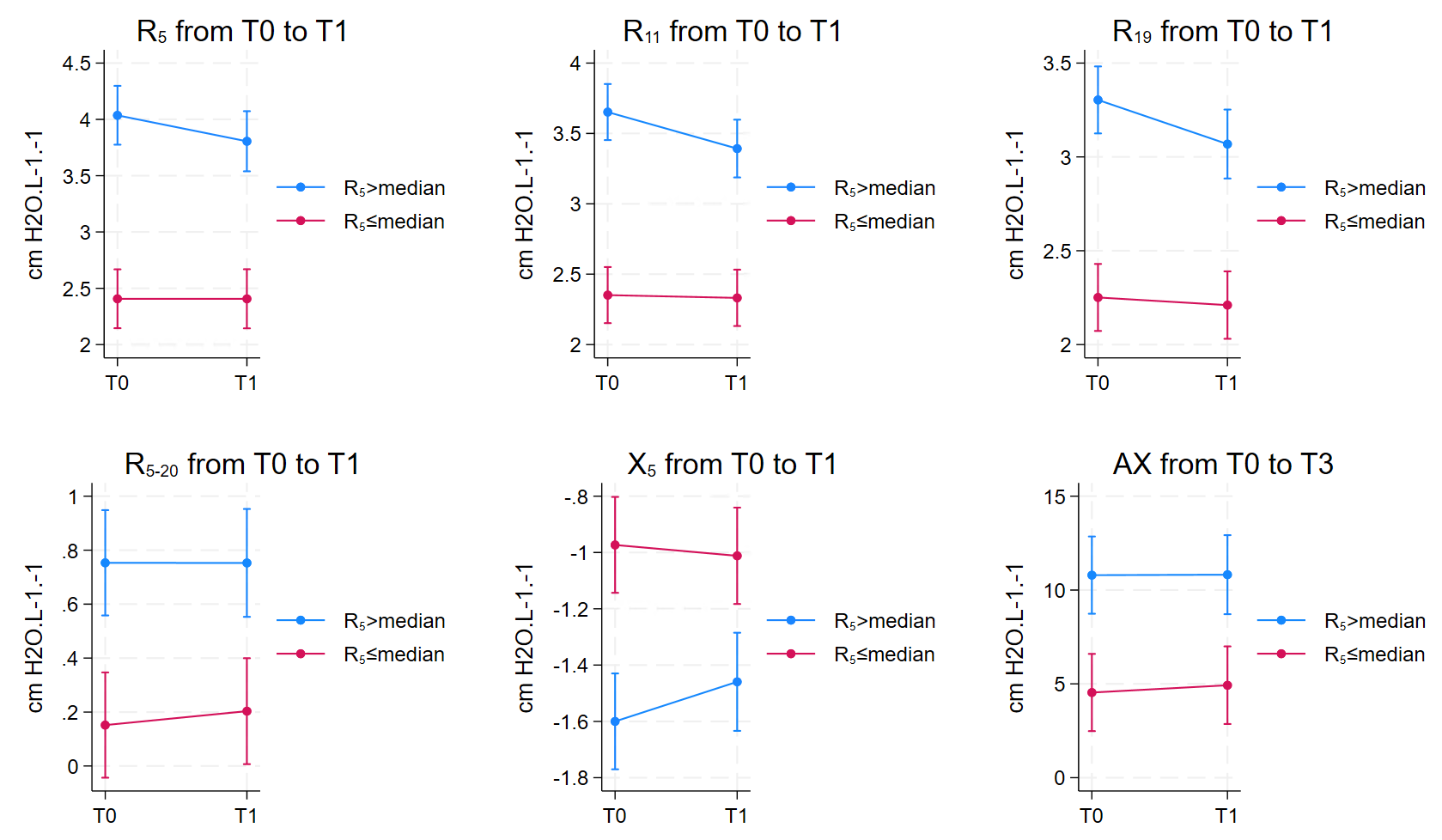


Supplementary figure 1 illustrates the changes in oscillometry variables from T0 to T1, stratified by patients with a baseline R_5_ above the median (blue line) and those with a baseline R_5_<median (red line). Graphs showing marginal means with 95% confidence intervals. The interaction term between group and time showed statistically significant differences for the resistance indices R_5_, R_11_, R_19_ and reactance measured at 5Hz (X_5_) (all p-values<0.05). Results from mixed effects model.

Supplementary table 1. Periodontal variables from baseline (T0) to six-week follow-up (T1).

|  | T0  *n*=57 | T1  *n*=57 | *p*-value |
| --- | --- | --- | --- |
| **Periodontal variables**  Mean BoP in % (SE)  Mean PI in % (SE)  Mean PD in mm (SE)  Mean CAL in mm (SE) | 63.10 (1.62)  62.00 (1.60)  2.49 (0.02)  2.72 (0.03) | 25.00 (1.62)  22.15 (1.60)  2.15 (0.02)  2.56 (0.03) | <0.001  <0.001  <0.001  <0.001 |

Supplementary table 1. Periodontal variables from baseline (T0) to six-week follow-up (T1). Data are presented as mean and standard error (SE). Abbreviations; BoP: bleeding on probing, PI: plaque index, PD: periodontal pocket depth, CAL: clinical attachment loss. Results from a mixed-effects model indicate the statistical significance of differences in means between timepoints, with baseline (T0) as the reference.

Supplementary figure 2. Alpha- and beta diversity at baseline, stratified by individuals above and below the baseline R_5_ median.


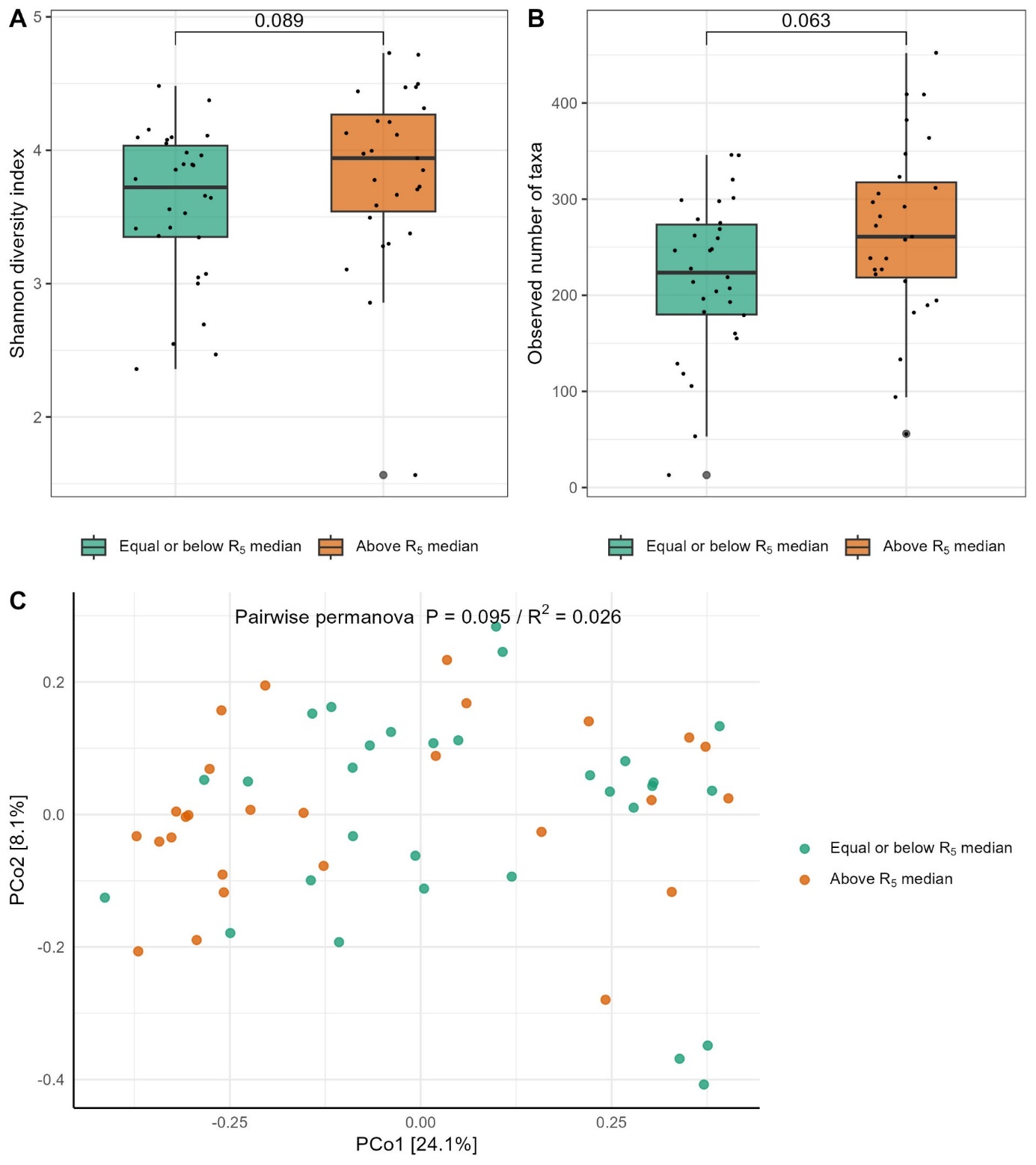


Supplementary figure 2. A and B: difference in baseline alpha diversity indices for baseline subgingival microbiome between individuals below the median of baseline R_5_ measurements (green boxes) and above (orange boxes); (A): Shannon diversity index; (B): Observed Number of taxa/MGS (Metagenomic species). Results from Wilcoxon rank-sum test *p*>0.05. (C) Principal coordinate analysis (PCoA) plot for comparison of baseline samples between individuals below the median of baseline R_5_ (green dots) and above (orange dots), (Bray-Curtis dissimilarity metric); pairwise PERMANOVA results, *p*=0.095. R^2^ represents proportion variance in the data explained by the grouping factor.

Supplementary figure 3. Change in periodontal parameters from T0 to T1 between individuals above and below the baseline R_5_ (absolute values) median.


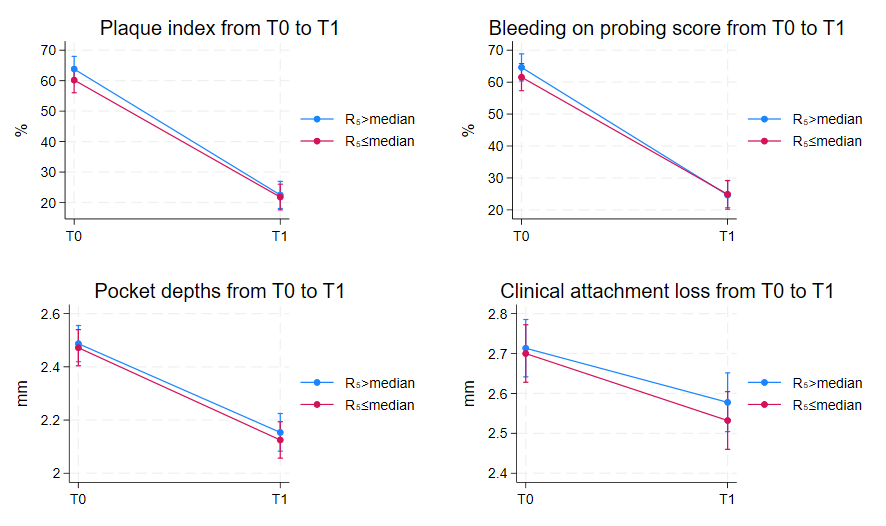


Supplementary figure 3 illustrates the changes in periodontal parameters from baseline (T0) to the six-week follow-up (T1) stratified by individuals above (blue) and below (red) the R_5_ median. Graphs showing marginal means with 95% confidence intervals. The interaction term between categories and time was not statistically significant for any of the parameters; BoP (bleeding on probing), plaque score, pocket depth, or clinical attachment level (all p-values>0.05). Results from mixed-effects model.

Supplementary figure 4. Alpha- and beta diversity at follow-up (T1), stratified by individuals above and below the baseline R_5_ median.


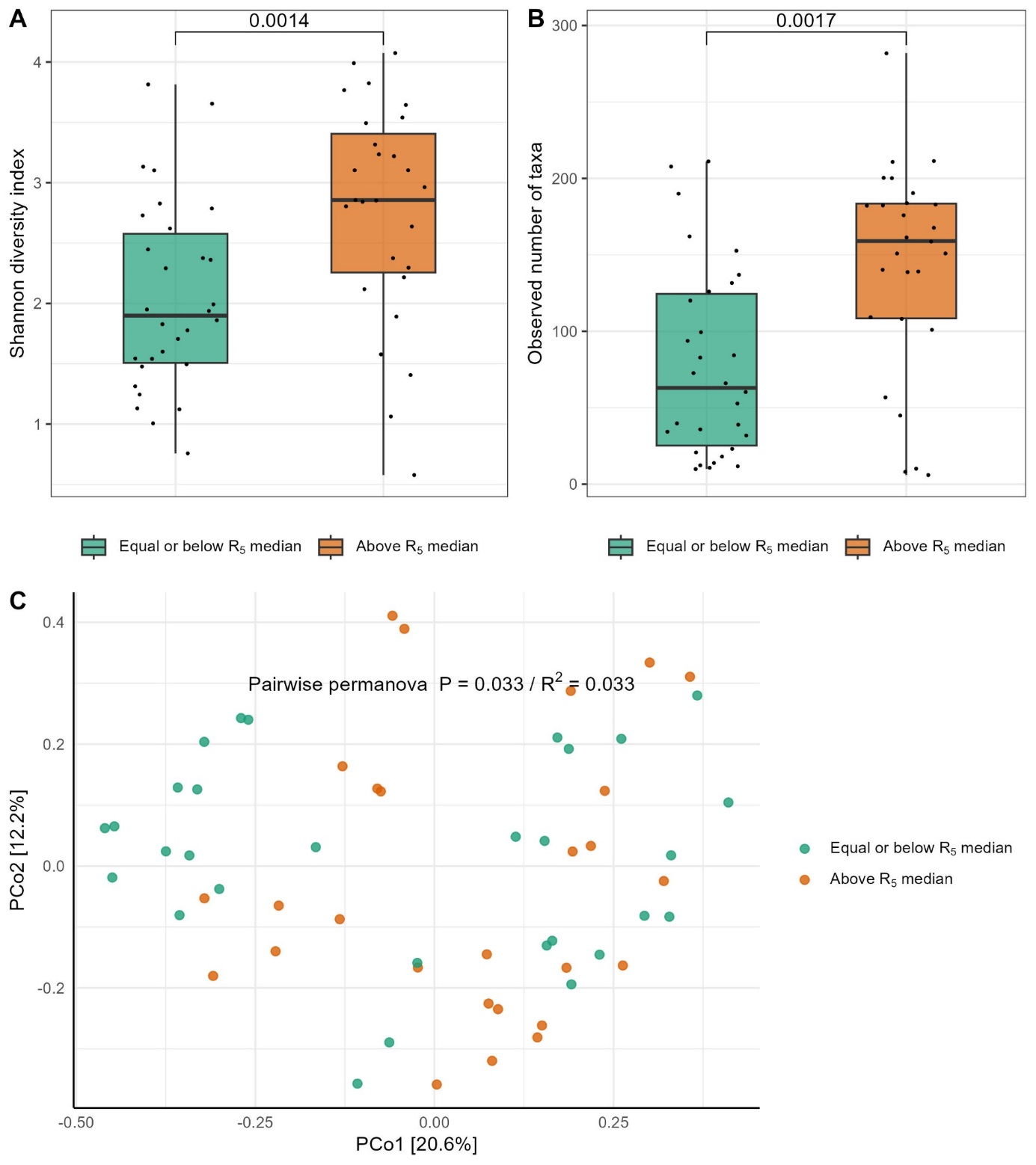


Supplementary figure 4. Difference in alpha and beta diversity indices for the follow-up subgingival microbiome between individuals below/equal to the median of R_5_ (green boxes) and above the R_5_ median (orange boxes); (A): Shannon diversity index; (B): Observed Number of taxa/MGS (Metagenomic species). Results from Wilcoxon rank-sum test *p*<0.01. (C) Principal coordinate analysis (PCoA) plot comparing follow-up samples between individuals above (orange dots) and below or equal to the R_5_ median (green dots) based on the Bray-Curtis dissimilarity metric. Pairwise PERMANOVA results, *p* = 0.033. R^2^ represents proportion variance in the data explained by the grouping factor.

# References

1. Nielsen HB, Almeida M, Juncker AS, Rasmussen S, Li J, Sunagawa S, Plichta DR, Gautier L, Pedersen AG, Le Chatelier E, et al: Identification and assembly of genomes and genetic elements in complex metagenomic samples without using reference genomes**.** *Nat Biotechnol* 2014, 32**:**822-828.

2. de Goffau MC, Lager S, Salter SJ, Wagner J, Kronbichler A, Charnock-Jones DS, Peacock SJ, Smith GCS, Parkhill J: Recognizing the reagent microbiome**.** *Nat Microbiol* 2018, 3**:**851-853.
